# Myocardial Damage by SARS-CoV-2: Emerging Mechanisms and Therapies

**DOI:** 10.3390/v13091880

**Published:** 2021-09-21

**Authors:** Huyen Tran Ho, Stefan Peischard, Nathalie Strutz-Seebohm, Karin Klingel, Guiscard Seebohm

**Affiliations:** 1 Cellular Electrophysiology and Molecular Biology, Institute for Genetics of Heart Diseases (IfGH), University Hospital Münster, 48149 Münster, Germany; Stefan.Peischard@ukmuenster.de (S.P.); Nathalie.Strutz-Seebohm@ukmuenster.de (N.S.-S.); 2 Cardiopathology, Institute for Pathology and Neuropathology, University Hospital Tübingen, 72076 Tübingen, Germany; karin.klingel@med.uni-tuebingen.de

**Keywords:** SARS-CoV-2, myocarditis, treatment of viral infection, myocardial damage, viral replication, viral docking, immune response, apoptosis, energy metabolism, viroporin

## Abstract

Evidence is emerging that severe acute respiratory syndrome coronavirus 2 (SARS-CoV-2) can infect various organs of the body, including cardiomyocytes and cardiac endothelial cells in the heart. This review focuses on the effects of SARS-CoV-2 in the heart after direct infection that can lead to myocarditis and an outline of potential treatment options. The main points are: (1) Viral entry: SARS-CoV-2 uses specific receptors and proteases for docking and priming in cardiac cells. Thus, different receptors or protease inhibitors might be effective in SARS-CoV-2-infected cardiac cells. (2) Viral replication: SARS-CoV-2 uses RNA-dependent RNA polymerase for replication. Drugs acting against ssRNA(+) viral replication for cardiac cells can be effective. (3) Autophagy and double-membrane vesicles: SARS-CoV-2 manipulates autophagy to inhibit viral clearance and promote SARS-CoV-2 replication by creating double-membrane vesicles as replication sites. (4) Immune response: Host immune response is manipulated to evade host cell attacks against SARS-CoV-2 and increased inflammation by dysregulating immune cells. Efficiency of immunosuppressive therapy must be elucidated. (5) Programmed cell death: SARS-CoV-2 inhibits programmed cell death in early stages and induces apoptosis, necroptosis, and pyroptosis in later stages. (6) Energy metabolism: SARS-CoV-2 infection leads to disturbed energy metabolism that in turn leads to a decrease in ATP production and ROS production. (7) Viroporins: SARS-CoV-2 creates viroporins that lead to an imbalance of ion homeostasis. This causes apoptosis, altered action potential, and arrhythmia.

## 1. Introduction

Currently, severe acute respiratory syndrome coronavirus 2 (SARS-CoV-2), which causes coronavirus disease 2019 (COVID-19), is threatening the entire world in one of the worst global pandemics, with over 200 million cases and an estimated 4.4 million deaths as of August 2021, and with new, more-threatening virus variants emerging according to the World Health Organization (WHO) [1].

**Figure 1:**
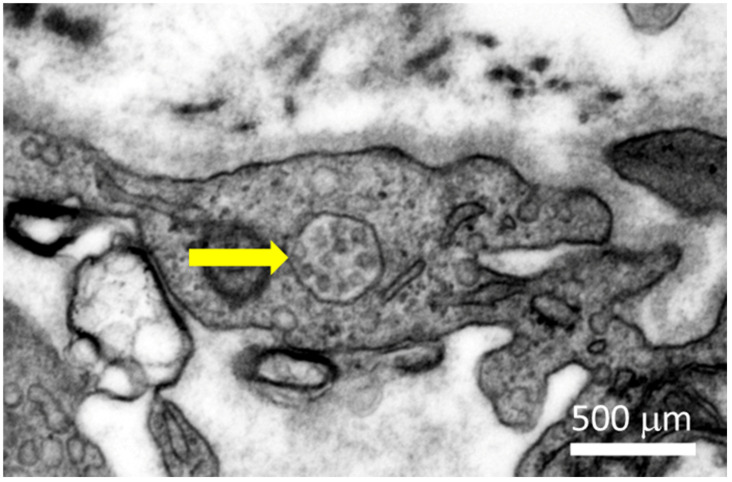
SARS-CoV-2 viral particles in an endothelial cell of the heart of a 64-year-old male COVID-19 patient. The yellow arrow indicates viral particles.

The virus mainly causes respiratory infection, which in severe cases causes viral pneumonia and acute respiratory distress syndrome (ARDS). Severe COVID-19 cases have been associated with myocardial injury, arrhythmia, acute coronary syndrome, venous thromboembolism, and micro- and macro-thromboses, which are associated with high mortality [2,3,4,5]. Myocarditis is defined as the inflammation in the myocardium. It is usually identified by immunohistochemical analysis of endomyocardial biopsies (EMBs). Characteristics include flulike symptoms, left ventricle systolic dysfunction, enhanced numbers of interstitial immune cells, and an increase in creatine kinase biomarker and cardiac troponin levels in the blood [6]. There have been cases of myocarditis in COVID-19 patients: Different studies have reported rates between 7.2% to 27.8% of COVID-19 patients developing cardiac issues. However, these studies did not elucidate whether the disease was caused by direct infection of the heart or indirectly (see also Section 2) [7,8,9,10,11,12,13,14]. There are studies that analyzed and detected SARS-CoV-2 presence in the heart. Interestingly, in some of these cases, no infection was found in nasopharyngeal swabs of patients. These findings were interpreted as indication of a primary cardiac infection [15,16,17,18,19]. Later, another study based on autopsies of deceased COVID-19 patients demonstrated that SARS-Cov-2 infection in the myocardium was common in these patients: 30 out of 41 patients were identified with SARS-CoV-2 in the myocardium. However, the number of infected cells in the myocardium was particularly low, and only four of the patients developed myocarditis. However, those patients with SARS-CoV-2 in the myocardium correlated with higher numbers of myocardial macrophages and lymphocytes, as well as cardiac inflammation and changes in the electrocardiographs [20]. Additionally, direct infection appeared to cause myofibrillar fragmentation in cardiomyocytes [21,22]. SARS-CoV-2 can infect endothelial cells and cause endotheliitis as well [23,24]. Electron microscopic imaging of a 64-year-old male COVID-19 patient showed viral particles in an endothelial cell of the heart (see Figure 1).

In conclusion, there are indications that SARS-CoV-2 can directly infect cardiomyocytes and cardiac endothelial cells. This poses a possible risk of causing myocarditis or other complex heart diseases. To this date, data for SARS-CoV-2 causing viral myocarditis is scarce and seems uncommon [25]. There were COVID-19 patients with myocarditis, but without SARS-CoV-2 directly present in the myocardium. This means that through other indirect mechanisms, SARS-CoV-2 can cause myocarditis as well [26]. In these cases, possible causes of myocarditis were a cytokine storm and a systemic inflammation in COVID-19 patients that causes autoimmune myocarditis [25,27].

SARS-CoV-2 mutant variants are emerging that have increased transmissibility and lead to higher mortality [28]. This is mainly due to mutations in the spike S protein, most commonly D614G; this leads to higher affinity of the spike protein to the host cell receptor angiotensin-converting enzyme 2 (ACE2), therefore facilitating viral entry. In addition, evasion of HLA-A24-mediated cellular immunity and potentially increased viral replication have been reported for recent mutants [29]. A study analyzing the effect of the variants B.1.1.7, B.1.351, and P.2 on different endothelial cell types in vitro showed higher viral uptake with these variants, especially in coronary artery endothelial cells. Furthermore, the variants had cytotoxic effects, in contrast to the wildtype SARS-CoV-2 [30].

It is important to evaluate how SARS-CoV-2 could affect the heart. This is the topic of this review. Other viruses frequently causing myocarditis, such as enteroviruses like coxsackievirus B3 (CVB3) that infect cardiomyocytes and parvovirus B19 (PVB19) that infects cardiac endothelial cells, are shown to hijack the biological processes of the host cell to their advantage while damaging the heart [31,32,33]. These include, among others, the immune system, viral replication, the autophagosomal pathway, cell death, energy metabolism, and ion homeostasis, which all play a significant role in myocardial function [31]. Hence, by recapitulating our current knowledge about the effects of well-known viruses like enteroviruses, which infect cardiomyocytes, or parvovirus B19, which infects endothelial cells, and with our understanding of similar coronaviruses during viral myocarditis progression, we can suggest potential actions of the new SARS-CoV-2 on the human heart. Several drug candidates targeting some of these mechanisms are in clinical trials, and are elucidated as possible treatment options against SARS-CoV-2 infection in the heart in this review. We included a glossary of key terms addressed in this review for easier understanding (see Table 1).

SARS-CoV-2 is an ssRNA(+) virus that belongs to the order *Nidovirales*, with a large genome size of approximately 30 kb [34]. It encodes structural proteins that include the spike (S protein), envelope (E), membrane (M), and nucleocapsid proteins (N). It has 14 open reading frames and polyproteins that are processed to 16 nonstructural proteins termed Nsp1 to Nsp16 [35,36]. SARS-CoV-1 shares around 77.1% of the protein sequence with SARS-CoV-2. Our understanding of SARS-CoV-1 can help in understanding analogous actions of SARS-CoV-2, as data for the new SARS-CoV-2 are still incomplete [37].

## 2. SARS-CoV-2 Causing Myocardial Damage Unrelated to Myocarditis

Myocarditis-like symptoms, including troponin rise and cardiac dysfunction, have also been observed without the presence myocardial inflammation in COVID-19 patients, namely in Takotsubo cardiomyopathy and type I and type II myocardial infarction. Takotsubo cardiomyopathy, also known as broken-heart syndrome, is triggered by a sudden extreme release of catecholamines under physical and emotional stress. High concentrations of catecholamines lead to β-adrenergic toxicity and calcium dysregulation in cardiomyocytes, which is essential for the excitation–contraction coupling. A hypothesis for the increase of Takotsubo cases in COVID-19 pneumonia patients is the interplay between catecholamine release and cytokine release from a dysregulated immune system (see also Section 6), which amplify each other in a positive feedback loop [38,39].

Type 1 myocardial infarction has also been reported in some patients [40]. Type 1 is an atherothrombotic coronary artery disease characterized by plaque rupture and thrombosis. Potential mechanisms include the activation of pathogen-associated molecular pattern (PAMP) receptors in immune cells in atherosclerotic plaques, leading to a higher risk for plaque rupture. PAMP recognition by the innate immune system can also result in coronary endothelial dysfunction. This in turn leads to vasoconstriction and thrombosis [41].

The cytokine storm leads to increase in oxygen consumption in the heart while also causing thrombosis and coronary heart spasm. This leads to a decrease in blood supply and to an imbalance in oxygen demand and supply in the heart. Consequently, this acutely damages the heart, also known as type 2 myocardial infarction [42].

## 3. Viral Entry via Spike S Protein

**Figure 2:**
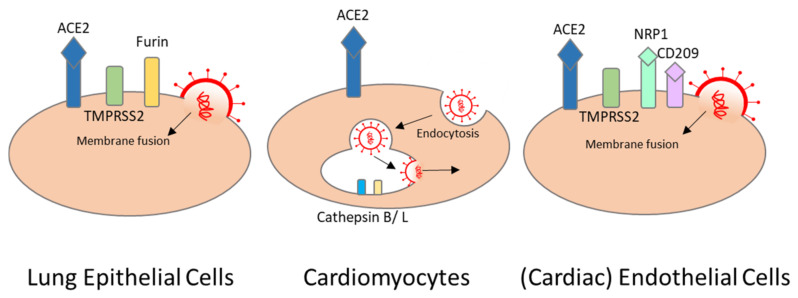
SARS-CoV-2 entry into cells of the heart. severe acute respiratory syndrome coronavirus 2 (SARS-CoV-2) can use angiotensin-converting-enzyme 2 (ACE2) as a receptor and different proteases to prime the spike protein. Depending on which protease is used for spike S protein priming, a different path is used for viral entry; i.e., membrane fusion or endocytosis. ACE2, angiotensin-converting-enzyme 2; TMPRSS2, transmembrane protease serine 2; NRP1, neuropilin-1.

SARS-CoV-2 binds to host cell surface proteins via the spike S protein. The host cell receptor is commonly formed by the angiotensin-converting enzyme 2 (ACE2) and by priming either the serine protease TMPRSS2, which mediates membrane fusion, or endosomal cathepsin B and L, which mediate viral entry via endocytosis [43,44]. In the lung, SARS-CoV-2 mainly targets type II pneumocytes that use ACE2 for binding, and TMPRSS2 and furin for priming [45]. Cardiomyocytes express high levels of ACE2 but extremely low levels of TMPRSS2. In cardiomyocytes, TMPRRS2 is localized in the cytoplasm and nucleus, rather than the plasma membrane where the enzyme is needed for priming [46,47]. However, high levels of cathepsin B and L that can compensate for TMPRSS2 are observed in cardiomyocytes. This would indicate endocytosis as the primary mechanism of SARS-CoV-2 entry [48] (Figure 2). Indeed, in vitro studies with hiPSC-cardiomyocytes and human-engineered heart tissues show that SARS-CoV-2 can directly infect these cells by entering via ACE2 and cathepsins, and that it can replicate in these cells [19,49]. Inhibition of cathepsin B and L via the inhibitor E-64d reduces SARS-CoV-2 infection significantly in iPSC-derived cardiomyocytes [21].

Nafamostat and camostat, both in clinical trials, are serine protease inhibitors that can potentially inhibit TMPRSS2, and thus viral entry. However, as cardiomyocytes do not express TMPRSS2, but instead cathepsin B and L, serine protease inhibitors that inhibit TMPRSS2 would most likely not be effective. Indeed, while cathepsin inhibitors are effective, the two TMPRSS2 inhibitors aprotinin and camostat mesilate did not have a significant effect in SARS-CoV-2-infected, iPSC-derived cardiomyocytes [21]. Hydroxychloroquine, among others, inhibits endosome-mediated viral entry of coronaviruses. It is therefore potentially effective against cathepsin-mediated virus entry [44]. However, there are concerns because it prolongs the QT interval and increases the risk of drug-induced sudden cardiac death. Later, it was dropped as a potential drug against SARS-CoV-2 due to low efficiency and the aforementioned risks [50,51,52]. In human-induced pluripotent stem cell (hiPSC)-derived cardiomyocytes, SARS-CoV-2 infection was successfully inhibited by the cathepsin inhibitor N-acetyl-l-leucyl-l-leucyl-l-methionine. Cathepsin inhibitors thus stand as a promising treatment option against SARS-CoV-2-infected hearts [49].

As previously mentioned, SARS-CoV-2 can infect endothelial cells, and can cause endotheliitis as well. Endothelial cells, especially vascular endothelial cells, abundantly express ACE2. Moreover, precursor endothelial cells express TMPRSS2. Nevertheless, it has been suggested that SARS-CoV-2 can also enter endothelial cells via neuropilin-1 and CD209L. Both are expressed abundantly in endothelial cells [53] (Figure 2). In line with this, in vitro studies showed significant decrease in infectivity using small-molecule inhibitors or antibodies against neuropilin-1, and should be considered as a treatment option that can potentially inhibit SARS-CoV-2 spike protein binding in endothelial cells [54,55].

There seems to be downregulation of ACE2 upon SARS-CoV-2 infection [56]. This could impact the human heart adversely, as ACE2 has a cardioprotective role and is relevant for cardiac contractility. It is part of the renin–angiotensin–aldosterone system (RAAS) that converts angiotensin I to angiotensin (1–7), which in turn promotes vasodilation [57,58,59]. Moreover, there are hints that ACE2 downregulation could lead to increased macrophage activation to promote inflammation [59]. ACE inhibitors (ACEi) and angiotensin-receptor blockers (ARBs) are used as treatment in many older patients with hypertension to regulate RAAS. These compounds enhance ACE2 expression. A meta-analysis of COVID-19 patients showed a tendency towards a decrease in deaths and critical events when using ACEi. Therefore, the use of ACEi and ARBs is evaluated to be safe in hypertensive patients during the pandemic, and might even be preferred in COVID-19 patients [60,61]. The S-mediated membrane fusion is inhibited by the pan-coronavirus fusion inhibitor EK1C4, which could represent a potent therapeutic option [62].

## 4. Viral Replication

**Figure 3:**
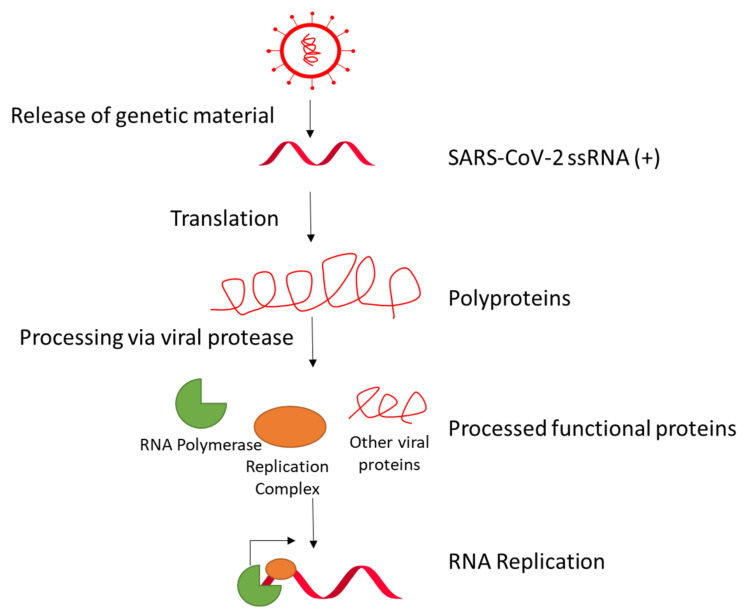
SARS-CoV-2 replication and viral protein translation. SARS-CoV-2 has ssRNA(+) as genetic material that is translated to polyproteins. The polyproteins are processed by viral proteases to generate more functional viral proteins; e.g., RNA-dependent RNA polymerase and replication complex for RNA replication.

Infected cardiomyocytes in COVID-19 patients are demonstrated to possess transcriptional activity [22]. Nsp1 in coronaviruses, including SARS-CoV-1, suppresses host gene translation by binding to the ribosome and promoting host mRNA degradation [63,64,65]. The other nonstructural proteins Nsp2 to Nsp16 comprise the viral replication and transcription complex (RTC), which include the RNA-processing and RNA-modifying enzymes and proteins for proofreading of the genome [66]. The reviews by V’kovski et al. and Romano et al. provide a good overview about the roles of the proteins in the SARS-CoV-2-RNA replication mechanism [36,67]. In brief, the positive-sense RNA can be immediately used for translating viral proteins (Figure 3). For RNA synthesis, the nascent positive strand is used to generate a negative-strand genomic RNA. This negative-stranded RNA is the template to generate the positive-stranded genomic RNA for the new virus particle, and to generate mRNA for translation of viral proteins. RNA replication is accomplished via discontinuous transcription, in which the RTC pauses at so-called transcription regulatory sequences (TRS). Transcription is performed until reaching a so-called body TRS (TRS-B) and can either continue to the next body TRS or jump to the end, where the leader TRS (TRS-L) is located and the last piece of mRNA is added. This leads to the creation of different-sized subgenomic mRNAs that translate into a plethora of different viral proteins and polyproteins. Further processing of the polyproteins is performed by viral proteases.

Viral RNA polymerase inhibitors, including remdesivir and favipiravir, have been evaluated in clinical trials as potential SARS-CoV-2 treatments. Remdesivir acts as a nucleoside analogue and is incorporated into the viral RNA product by the viral RNA-dependent RNA polymerase [68]. Remdesivir showed no significant effect during clinical trials, but is still FDA-approved as a drug against SARS-CoV-2 [52,69]. However, when remdesivir was used in hiPS-cell-derived cardiomyocytes and EHTs, SARS-CoV-2 infection was successfully inhibited [19,49]. The 3C-like protease encoded by nsp5, along with papain-like proteinase encoded by nsp3, cleave polyprotein precursors to form the replication complex for viral replication. Lopinavir/ritonavir are protease inhibitors and can potentially inhibit viral 3C-like protease, but clinical trials so far have been discouraging [70].

## 5. Autophagy and Double-Membrane Vesicles

Autophagy is an intracellular process to recycle misfolded proteins and organelles, and eliminate intracellular pathogens in autophagosomes as an innate immune response [71]. However, several positive-stranded RNA viruses such as CVB3 can manipulate autophagosomes by inhibiting lysosome–autophagosome fusion, which prevents virus elimination [72,73,74]. The autophagosmal pathway appear to be relevant for viral replication: Enteroviruses misuse autophagosomes as replication organelles, or use parts of the autophagosomal pathway for replication [75,76,77].

**Figure 4:**
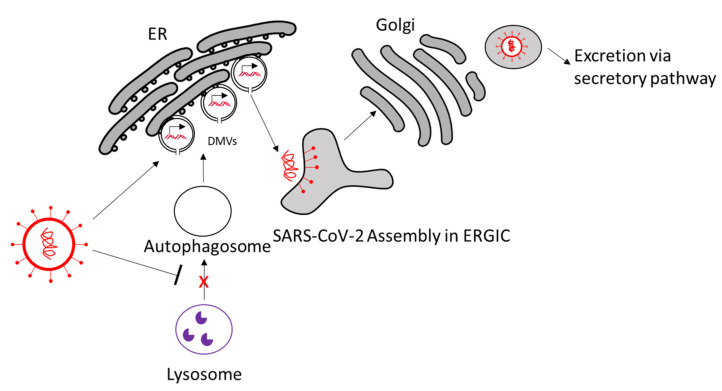
SARS-CoV-2 uses double-membrane vesicles and the autophagosomal pathway for replication. SARS-CoV-2 inhibits the fusion of lysosomes with autophagosomes. SARS-CoV-2 uses DMVs originated from the ER as replication sites and uses parts of the autophagosomal pathway for replication. Viral particles are assembled in the ERGIC and are excreted via the secretory pathway. ER, endoplasmic reticulum; DMV, double-membrane vesicle; ERGIC, ER–Golgi intermediate compartment.

Autophagosomes are manipulated in SARS-CoV-2 infection as well (Figure 4). Analogous to CVB3, Nsp3a of SARS-CoV-2 has been shown to inhibit lysosome–autophagosome fusion by disrupting Rab7–HOPS complex formation, which is needed for lysosome–autophagosome fusion. This leads to blockage of autophagy and accumulation of autophagosomes [78]. The use of autophagy-inducing compounds has been shown to inhibit SARS-CoV-2 propagation in vitro, making autophagy induction an interesting treatment option [79].

Accumulation of nonlytic autophagosomes can be beneficial for SARS-CoV-2 by using them as autophagosome-like double-membrane vesicles (DMVs). Indeed, these DMVs are induced in SARS-CoV-2-infected cells, described as inhabiting double-stranded RNA, and originate from the ER [80]. Coronaviruses can abuse components that are used for formation of vesicles for ER degradation called EDEMosomes to promote DMV formation [81]. DMVs of coronaviruses are in a closed conformation and connected to the ER [82,83,84]. Nsp3, -4, and -6 appear to be relevant for DMV formation in SARS-CoV-1-infected cells [85]. To transport the newly synthesized viral RNA from the vesicle to the cytosol, coronaviruses create molecular pores in the closed DMVs. The pore is potentially composed of nsp3 and possibly -4 and -6, all of which are transmembrane proteins [86].

Hence, targeting DMV formation could be a promising approach against SARS-CoV-2 infection and should be further studied. For example, the inhibition of cytosolic phospholipase A2 and early secretory protein GBF1 reduced DMV numbers and virus replication [87,88]. Other DMV inhibitors that are found for analogous viruses could be studied as potential SARS-CoV-2 treatment [74]. After viral RNA export from the DMVs, the viral RNA, along with viral structural proteins, are assembled in single-membrane compartments made from the ER-to-Golgi intermediate compartment (ERGIC), and the newly assembled virions are excreted via the secretory pathway into the extracellular space [86,89,90].

## 6. Immune Response

**Figure 5:**
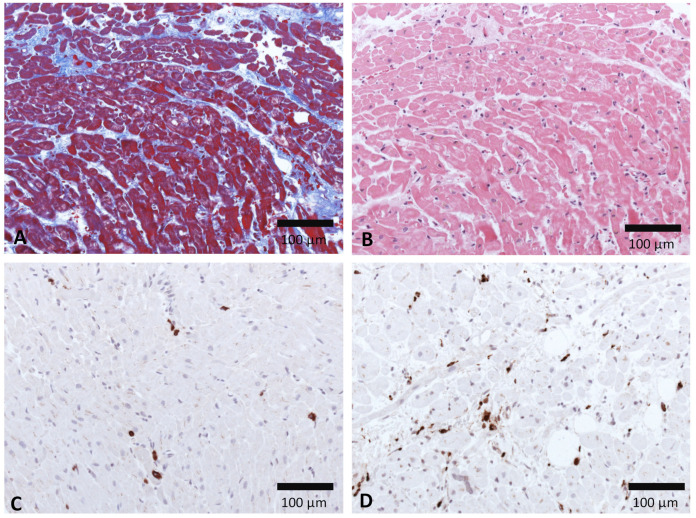
Healing myocarditis in a SARS-CoV-2 positive patient (75 years old, male). (**A**) Masson Trichrome (**B**) H&E, (**C**) CD3+ T cells, (**D**) CD68+ macrophages of the interstitium (bar = 100 μm).

There are indications that the immune system contributes to the pathology of SARS-CoV-2 myocarditis and myocardial injury. Cytotoxic T-lymphocytes and macrophages are reported to infiltrate the myocardium in SARS-CoV-2-induced myocarditis patients [16,17,18]. As exemplarily shown in Figure 5, EMBs from patients with COVID-19 revealed more CD68+ macrophages and a few CD3+ T cells. Patients with severe COVID-19 disease progression have increased IL-17 secreting T-helper 17 (TH17) cells [91]. Cytotoxic T cells and macrophages in CVB3-induced viral myocarditis are described to promote cardiac damage. Studies indicate that there is a major role for TH17 in promoting progression towards dilated cardiomyopathy and heart failure [92,93,94]. Thus, a SARS-CoV-2 infection in the heart could cause analogous adverse immune responses leading to myocarditis, DCM, or heart failure. Humoral and cellular immune signatures that can predict a risk for progression towards heart diseases, including further knowledge of heart-reactive autoantibodies present during SARS-CoV-2 infection, should be further elucidated [95].

Two stages during COVID-19 disease progression have been described: the early stage, which is more focused on immune protection, viral replication, and apoptosis inhibition; and the later severe stage, in which the cytokine storm is described to damage the tissue due to inflammation [96]. In the myocardium, Lindner et al. reported an increase of cytokine expression, including TNF-α; IFN-γ; chemokine ligand 5; and IL-1β, -6, -7, -8, and -18 [97]. Inflammasomes contain pattern-recognition receptors, including NLR family pyrin domain containing 3 (NLRP3), and inflammatory procaspases, including procaspase-1. They contribute to the maturation of proinflammatory cytokines such as IL-1β and induction of pyroptosis [98]. NLRP3 inflammasomes are shown to be elevated by the SARS-CoV-1 nsp3a, -8b, and E proteins, which promote inflammation reactions [99,100,101]. The structural proteins of SARS-CoV-1, as well as nsp1, -3a, and -7a, appear to be able to enhance nuclear factor-κB (NF-κB) signaling. The increase in NF-κB signaling could be responsible for the cytokine storm seen in the later severe stages of COVID-19 [96,102]. Inhibition of NF-κB in SARS-CoV-1-infected mice did not affect virus titers, but decreased inflammatory cytokines, namely TNF, CCL2, and CXCL2 in combination with higher observed survival rates [103].

**Figure 6:**
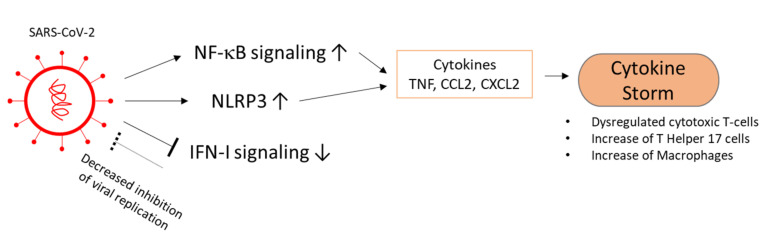
SARS-CoV-2 manipulates and dysregulates immune response. The dotted arrows indicate a decreased effect through viral effect. NF-κB, nuclear factor-κB, NLRP3, NLR family pyrin domain containing 3; IFN-I, interferon type I; TNF, tumor necrosis factor; CCL2, chemokine (C-C motif) ligand 2; CXCL2, chemokine (C-X-C motif) ligand 2.

Coronaviruses interfere with interferon type I (IFN I) induction and signaling in early stages. In agreement with this, SARS-CoV-2 patients do not show high levels of IFN-I response. Impaired or delayed IFN-I response promotes progression towards COVID-19 with accumulation of monocytes–macrophages, increased viral replication, hypercytokinemia, or cytokine storm with a dysregulated T-cell response (see Figure 6) [104,105]. Thus, treatment with IFNs can be beneficial against SARS-CoV-2, but should be applied in early stages. Late administration of IFN I is shown to be ineffective or have adverse effects in animals with SARS-CoV-1 or MERS-CoV infection [104]. In an in vitro study with SARS-CoV-2-infected, hPSC-derived cardiomyocytes and EHTs, elevation of cytokines and chemokines, including IFN I signaling and TNF expression, was observed. In addition, macrophages accumulated in the sites of SARS-CoV-2 infection in the EHT model, indicating that cardiomyocyte infection leads to macrophage activation [19]. It must be further elucidated what role IFN I signaling has in coronavirus induced myocarditis, as IFN I response varies between different cell types and within different microenvironments [104,106].

In general, suppression of certain transcription factors or proinflammatory cytokines such as NF-κB; e.g., with proteasome inhibitors in later-stage infections, and IFN I administration during early infection stages, could be beneficial in COVID-19 [107,108]. Immune modulation via interferons such as IFN-β1a, IFN-α, PegIFN-α2β, IFN-α1β, and IFN-β1β have been evaluated in clinical trials as adjuvants against SARS-CoV-2 [69]. However, interferon IFN-β1a was already dropped from trials because the drug did not show significant effects in patients [52]. In this study, patients that were hospitalized with COVID-19 were treated with IFN-β1a, therefore similar to SARS-CoV-1 and MERS-CoV, it could be ineffective due to late administration. A triple combination of IFN-β1β with lopinavir–ritonavir and ribavirin was shown to have a beneficial effect on COVID-19 patients in a phase 2 study [109]. IFN-β administration in patients with enteroviral or parvovirus-B19-induced myocarditis in clinical studies showed to be safe and have ameliorating effects, so interferon treatment in myocardium SARS-CoV-2 myocarditis may be promising [110,111,112].

## 7. Programmed Cell Death

**Figure 7:**
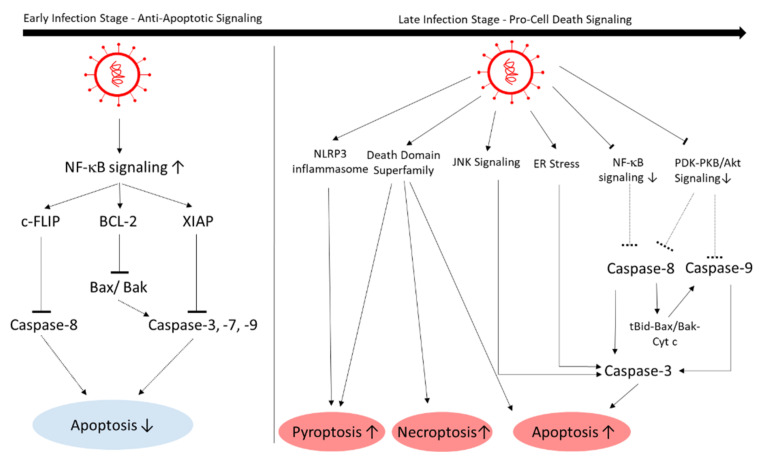
SARS-CoV-2 manipulates cell death. In early infection stages, cell death is inhibited to promote viral replication, while in later infection stages, cell death is promoted to enhance viral spread. The dotted arrows indicate a decreased effect through viral effect. C-FLIP, cellular FLICE (FADD-like IL-1β-converting enzyme)-inhibitory protein; BCL-2, B-cell lymphoma 2; XIAP, X-linked inhibitor of apoptosis protein, Bax, Bcl-2-associated X protein, Bak, BCL-2 antagonist/killer-1; ER, endoplasmic reticulum; JNK, c-Jun N-terminal kinase; NF-κB, nuclear factor-κB; PDK, 3-phosphoinositide-dependent kinase; PKB/Akt, serine/threonine kinase protein kinase B; tBid, truncated BH3 interacting-domain death agonist; Cyt c, cytochrome c; NLRP3, NLR family pyrin domain containing 3.

To efficiently replicate, cardiogenic viruses such as enteroviruses inhibit apoptosis signaling at the beginning of viral infection. In later stages, the focus shifts to viral release, so enteroviruses manipulate the host cell towards apoptosis [113]. PVB19 utilizes its nonstructural protein NS1 to trigger cell-cycle arrest to induce apoptosis in endothelial cells in the late viral cycle [33,114]. Similar to enteroviruses, SARS-CoV-2 also blocks apoptosis in earlier stages and induces cell death in later stages [115] (Figure 7). An in vitro study with EHT showed cell death as a result of SARS-CoV-2 infection and not inflammation [19].

In early stages, NF-κB signaling is upregulated, which is an important antiapoptotic pathway. Activated NF-κB upregulates apoptosis inhibitors such as cellular FLICE (FADD-like IL-1β-converting enzyme)-inhibitory protein (c-FLIP), Bcl-2 family members, and the X-linked inhibitor of apoptosis proteins (XIAPs) [115].

In later stages, in which the cytokine storm occurs, apoptosis, necroptosis, and pyroptosis are initiated via different pathways. For instance, in contrast to earlier stages, in later stages NF-κB is suppressed by the SARS-CoV-1 M protein, which leads to decreased apoptosis inhibition [116]. In the lungs of a transgenic mouse model, apoptosis and necrosis was induced via caspase-8 activation [117]. The Nsp3, -6, -7, and -8 proteins also appear to play a role in inducing apoptosis: The 3a protein of SARS-CoV-2 was shown to induce apoptosis in HEK293T, HepG2, and Vero E6 cells. Nsp3a cleaves and activates caspase-8. The active caspase-8 then activates the extrinsic apoptotic pathway. Further, SARS-CoV-2 increases and cleaves Bid to tBid, which activates Bax/Bak, cytochrome c, and caspase-9, which in turn activates the mitochondrial intrinsic apoptotic pathway [118]. Thus, it seems likely that caspase-8 localizes to the mitochondrial membrane and cleaves Bid to initiate the intrinsic apoptotic pathway [119]. The Nsp7a protein appears to inhibit host-cell protein synthesis, possibly as a host-cell stress response, and elevates p38 mitogen-activated protein-kinase (MAPK) signaling, which induces apoptosis [120]. SARS-CoV-1 Nsp6 induces caspase-3-mediated, ER-stress-induced, and JNK-dependent apoptosis [121]. Nsp8b promotes cell death by forming intracellular aggregates. The aggregates causes ER stress in a caspase-independent way, and activates NLRP_3_ inflammasomes, which then activate inflammation reactions and pyroptosis, another form of cell death [100]. There are indications that the structural proteins E, S, and M regulate apoptosis as well: The E protein of SARS-CoV-1 induces T-cell-mediated apoptosis, which in turn can be inhibited by overexpressing Bcl-xL [122]. The S protein alone is able to induce apoptosis, but its mechanism remains elusive [123]. The M protein activates apoptosis by interfering with the PDK1-PKB/Akt signaling, thus activating caspase-8 and -9 [124]. The death domain superfamily also plays a complex role in regulating pyroptosis, necroptosis, and apoptosis in SARS-CoV-2 infection; this has been thoroughly described by Ivanisenko et al. [115].

In summary, SARS-CoV-2 infection leads to suppression and induction of cell death by several viral proteins. There seems to be a pattern of time-dependent manipulation of apoptosis, in which extrinsic apoptosis is inhibited at early stages and apoptosis and pyroptosis are prevalent in later, more severe stages that promote inflammation. However, inhibition of caspase-mediated apoptosis using Bcl-2 in SARS-CoV-1-infected Vero cells showed no significant change in susceptibility to infection, kinetics, viral replication, or release, which poses a question regarding what role apoptosis plays in SARS-CoV-2 pathogenesis [125]. The use of anti-inflammatory treatment strategies and targeting components of the death domain superfamily are promising [115]. Moreover, the regulation of apoptosis seems to be dependent on the cell type, thus cell death in SARS-CoV-2-infected hearts must be further elucidated [126].

## 8. Energy Metabolism

During viral myocarditis, energy metabolism is disturbed, which can lead to adverse effects on myocardial performance and cell-damaging ROS production. Consistently, disturbed energy metabolism indicates severe disease progression. Mice with CVB3-induced myocarditis have reduced mitochondrial ATP/ADP ratios, and the activity of the respiratory chain (RC) in mitochondria has been shown to be decreased or imbalanced [127]. Interestingly, imbalanced RC activity that leads to high ROS production and apoptosis in mice correlates with efficient viral elimination, while overall decrease of RC activity correlates with persistent infection [128]. High ROS production is also observed in human-iPSC-derived cardiomyocytes that express CVB3 [129].

CVB3 in murine atrial cardiomyocytes can stimulate dynamin-related protein 1 (Drp1) by localizing itself to mitochondria, which leads to mitochondrial fragmentation, producing extracellular membrane vesicles. CVB3 can utilize these vesicles to release viral particles [130]. Inhibition of Drp1 has been shown to prevent mitochondrial damage and myocardial injury in CVB3-induced myocarditis [131].

**Figure 8:**
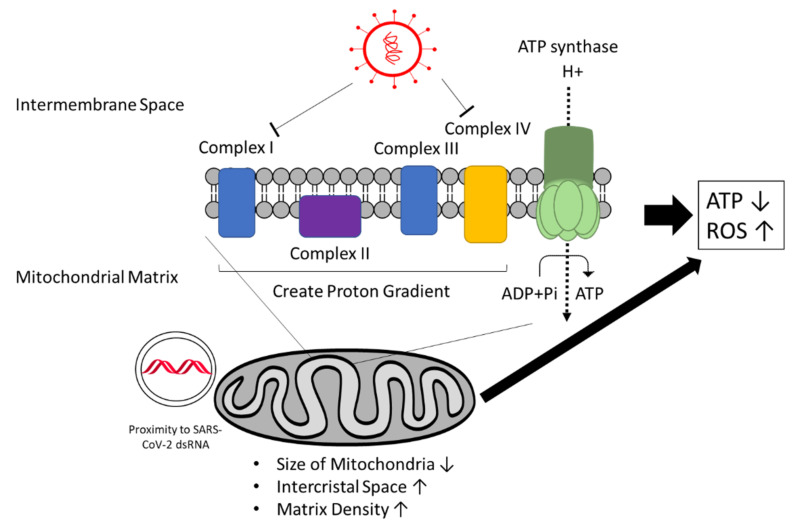
SARS-CoV-2 manipulates energy metabolism. SARS-CoV-2 affects the morphology of mitochondria and components of the respiratory chain. A decrease in ATP and an increase in ROS production is observed, which could be the result of altered mitochondria function and respiratory chain activity. ATP, adenosine triphosphate; ROS, reactive oxygen species.

Mitochondria and energy metabolism are affected by SARS-CoV-2 as well (Figure 8). Computational modeling predicts SARS-CoV-2 RNA to localize to the mitochondria, which may impact mitochondrial function [132]. Indeed, mitochondrial accumulation has been observed using integrative imaging around the viral replication sites where dsRNA is present. Furthermore, the mitochondria appear smaller in size, and intracristal space and matrix density are increased in infected cells. In addition, a decrease was reported in ATP synthase subunit 5B associated with a decrease in ATP production [84]. SARS-CoV-2 also appears to downregulate mitochondrial proteins such as NDUFA10, a component of the mitochondrial complex I, as a master regulator [133]. Moreover, interactions of viral proteins with other components of complex I and complex IV have been observed, which could lead to imbalanced RC complex activity and ROS production [134,135,136,137].

A recent study by Ajaz et al. analyzed mitochondrial respiration in peripheral blood mononuclear cells from COVID-19 patients and described reduced ATP-linked respiration, reserve capacity, and maximal respiration, indicating compromised mitochondrial respiration or mitochondrial dysfunction. In addition, increased glycolysis was observed, which could compensate for the decreased respiration [138]. Therefore, the virus seems to depend more on glycolysis for energy production.

Altogether, cardiogenic viruses such as CVB3 and SARS-CoV-2 alter mitochondrial structures and mitochondrial respiration. SARS-CoV-2 likely manipulates mitochondrial function to suppress antiviral response by the host immune system [139]. The resulting impairment of energy metabolism and ATP production can cause reduction of cardiac function [140]. Furthermore, it is assumed that ROS are increasingly produced, especially in severe cases of COVID-19 due to hypoxia, the cytokine storm, and dysfunction of mitochondria. This would lead to oxidative stress and promote inflammation [141]. Higher ROS levels may lead to more apoptosis, and thus loss of functional cardiomyocytes and the spread of viral particles. On the other hand, more apoptosis can also lead to more efficient viral clearing and prevention of chronic disease development: High apoptosis rates correlated with functional recovery in myocarditis patients, while inhibition of apoptosis correlated with chronic myocarditis [142,143].

## 9. Viroporins

Viroporins are viral proteins that are similar to ion channels; they both integrate into the membrane of host cells and conduct ions. Most animal RNA viruses form these viroporins [144,145,146]. The main functions of viroporins are to aid virion assembly and release from the host cell. By altering the ion concentration gradient, viroporins can cause depolarization that supports the budding of the virus. Furthermore, viroporins localize at the budding neck, the part of the cellular membrane that constricts and forms the budding enveloped virion. At this location, the viroporins can oligomerize to facilitate virion scission [147]. Further supported functions include cell entry, genome replication, and manipulation of programmed cell death. As a consequence, the host-cell ion concentration; the membrane permeability of ions; and the activity of ion channels that conduct ions such as Na^+^, K^+^, and Ca^2+^ are altered. As a counteraction, the host immune system can sense the intracellular K^+^ and Ca^2+^ imbalances caused by viroporins and activate NLRP3 inflammasomes in several respiratory RNA virus infections [146].

Enteroviruses, including CVB3, form a Ca^2+^-conducting viroporin that alters Ca^2+^ homeostasis. This viroporin inserts itself into the plasma membrane, as well as the membranes of mitochondria, the Golgi apparatus, the ER, and the SR. This leads to apoptosis suppression and induction in a time-dependent manner [148]. PVB19 does not seem to possess a viroporin, but the virus causes endothelial dysfunction by inducing Ca^2+^ entry via host ion channels, which disturbs the ion homeostasis [33].

**Figure 9:**
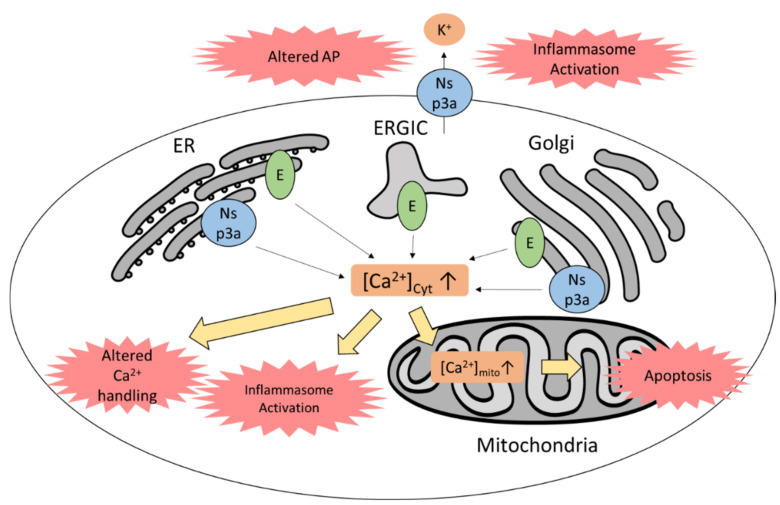
SARS-CoV-2 proteins form cationic viroporins, causing inflammasome activation, altered action potential and Ca^2+^ handling, and apoptosis. ER, endoplasmic reticulum; ERGIC, ER–Golgi intermediate compartment; AP, action potential; cyt, cytosolic; mito, mitochondrial.

Interestingly, SARS-CoV-1 also creates viroporins from the structural proteins E, nsp3a, and -8a (Figure 9). Proteins E and nsp3a share over 85% similarity with the analogous proteins of SARS-CoV-2 [149,150]. The E protein is shown to be a cationic viroporin for Na^+^, K^+^, and Ca^2+^, being more selective for Na^+^ and Ca^2+^, which localize to the ER, Golgi apparatus, and the ER–Golgi compartment (ERGIC) [101,151,152,153]. Nsp3a forms a cationic viroporin at the plasma membrane, ER, and Golgi that conducts Na^+^, K^+^, and Ca^2+^, with selectivity for K^+^ and Ca^2+^ [149,154,155,156]. Thus, there is a possibility that the E protein and Nsp3a increase cytosolic Ca^2+^ concentration by releasing Ca^2+^ from the intracellular stores, leading to Ca^2+^ overload, Ca^2+^ uptake in mitochondria, and thus apoptosis. Both viroporins appear to be relevant for virus viability and replication. Moreover, it was shown that both E protein and Nsp3a cause NLRP3 inflammasome activation by causing Ca^2+^ or K^+^ efflux, respectively [101,157]. Nsp8a, on the other hand, forms a weak cation-conducting viroporin, and does not appear to play a major role in virulence, unlike the E protein and Nsp3a [149,158].

A computational model of human ventricular myocytes indicated that SARS-CoV-2 viroporin activity can dysregulate action potential and Ca^2+^ handling, as the E protein and Nsp3a have similar channel properties as the outward K^+^ current (*I_to_*) in cardiomyocytes [150]. A SARS-CoV-2 infection in the heart could hence lead to arrhythmia. Suppressing SARS-CoV-2 viroporin activity thus poses an attractive treatment option against SARS-CoV-2 myocarditis and other diseases caused by viroporin-forming viruses. Adamantanes are able to inhibit several viroporins. They are shown to be effective against the SARS-CoV-1 E protein, and a case report on SARS-CoV-2 patients indicated that the use of adamantanes could be protective against progression to COVID-19 [159,160]. Hexamethylene amiloride is also able to inhibit the E protein, resulting in reduced viral replication in several coronaviruses [161]. In patients with viral diseases including severe COVID-19, hypocalcemia, a decrease in serum Ca^2+^, has been observed. Hypocalcemia is assumed to be a host defense mechanism against increasing intracellular Ca^2+^ concentrations caused by viruses [162]. Decreasing intracellular Ca^2+^ can thus be effective against SARS-CoV-2: calcium chelators or calcium inhibitors can be used in reducing inflammasome activation. Whether this can reduce viral replication in SARS-CoV-2 infection has yet to be elucidated, but it is known to have no effect on replication of rhinovirus, which expresses viroporin 2B [163]. Calcium channel blockers can also be considered as a treatment against SARS-CoV-2, as they can suppress viral entry and have anti-inflammatory effects in SARS-CoV-1 or Mers-CoV infections [162].

## 10. Summary

**Figure 10:**
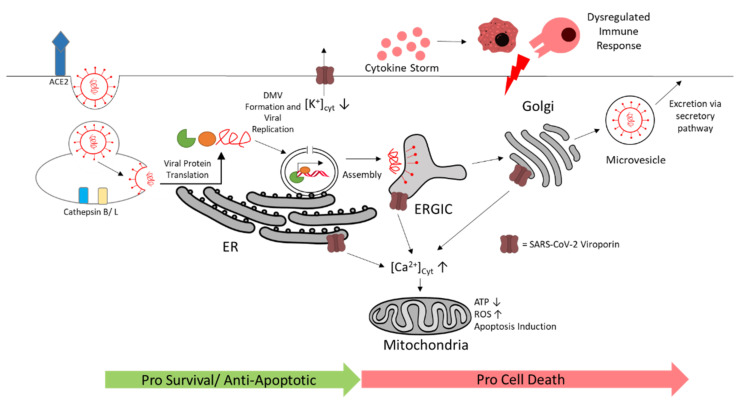
SARS-CoV-2 virus–host interactions. ACE2, angiotensin-converting enzyme 2; ER, endoplasmic reticulum; ERGIC, ER–Golgi intermediate compartment; ATP, adenosine triphosphate; ROS, reactive oxygen species.

In this review, we explained SARS-CoV-2 entry in cardiac cells, the replication mechanism and manipulation of autophagy and DMVs for viral replication, SARS-CoV-2 interaction with the immune system, manipulation of programmed cell death, effects on host energy metabolism, and myocardial effects by viroporins expressed by SARS-CoV-2 (see Figure 10). Several of these virus–host interactions for SARS-CoV-2 are analogous to well-known cardiogenic viruses like the ssRNA(+) virus CVB3 and ssDNA virus PVB19 (see Table 2).

To date, the incidence rate of SARS-CoV-2-induced myocarditis has not been determined due to incomplete data. Still, there is a possibility that SARS-CoV-2 has cardiovascular effects, and a high possibility that direct myocardial infection can occur. Myocardial damage caused by SARS-CoV-2 also can become an emerging issue in regard to new mutations of SARS-CoV-2 that were shown to have increased virulence. SARS-CoV-1 and -2 appear to have similar effects on the heart as other myocarditis-causing viruses, but further studies of SARS-CoV-2 in the heart have to be performed. Several antiviral drugs against SARS-CoV-2 infections are in clinical trials, but several have already been found to be ineffective [52,69]. The efficacy of these drugs on SARS-CoV-2 infections in the heart still must be further elucidated. Some of these viral therapies are considered potentially cardiotoxic, and drug-induced myocarditis could occur [25].

## Figures and Tables

**Table 1:** Glossary of key terms used in this review.

Key Term	Definition
Cardiomyocytes	Heart muscle cells. Responsible for mechanical contractions.
Endothelial cells	Cells that form the interior wall of blood vessels.
Inflammation	Local response of the body against (alleged) foreign bodies such as pathogens. In this process, the immune system tries to remove the pathogens, which can be harmful when the reaction is excessive. Characteristic reactions are heat, pain, swelling due to capillary dilation, and leukocyte infiltration.
Myocarditis	Inflammation of the myocardium. Characteristics include flulike symptoms, left ventricle systolic dysfunction, enhanced numbers of interstitial immune cells, and an increase in creatine kinase biomarker and cardiac troponin levels in the blood.
Endotheliitis	Inflammation of the endothelium in the blood vessels.
Cytokine storm	Reaction by the innate immune system in which an excessive amount of proinflammatory signals; i.e., cytokines, are released.
Spike S protein	A specific protein structure of SARS-CoV-2 located on the surface of the viral envelope. It is used by the virus to dock onto the host cell.
Angiotensin-converting-enzyme 2 (ACE2)	An enzyme located at the surface of specific cells. It catalyzes angiotensin II to angiotensin (1–7), which leads to vasodilation.
Viral protease	Proteins expressed by the virus that catalyze the cleavage of specific proteins. Viral proteases are usually used to process their own precursor polyproteins and manipulate host proteins.
Autophagy	Cell mechanism to remove misfolded proteins, organelles, and pathogens within the cell. This process usually includes the formation of vesicles called autophagosomes as the site for digestion.
Double-membrane vesicle	Vesicles that appear to have two membranes made of components of intracellular organelles such as the ER, ERGIC, or Golgi. They are created and used by specific RNA viruses as a site of replication.
Inflammasome	Oligomers secreted by the innate immune system that activate specific inflammatory reactions.
Apoptosis, necrosis, and pyroptosis	Apoptosis is the process of programmed cell death. The apoptotic cell creates cell fragments that are removed by phagocytes. Necrosis describes an uncontrolled cell death in which the membrane ruptures and intracellular contents are released. Pyroptosis is also a form of programmed cell death that is triggered by inflammatory reactions.
Respiratory chain	Known additionally as the electron-transport chain. It is composed of 5 protein complexes in mitochondria, transports electrons and creates a proton gradient to generate ATP.
Viroporin	A viral protein that can selectively conduct ions similar to ion channels. They promote viral replication and manipulate biological processes in the host cell.

**Table 2:** Comparison of several virus–host interactions and viral mechanisms between SARS-CoV-2, CVB3, and PVB19 in myocarditis.

	SARS-CoV-2	CVB3	PVB19
Viral Genome	ssRNA(+) [34]	ssRNA(+) [164]	ssDNA [165]
Infected cardiac cell types	- Cardiomyocytes [22,49] - Cardiac endothelial cells [23,24]	Cardiomyocytes [166]	Cardiac endothelial cells [167]
Receptors/primers for viral entry	- ACE2 [48,53] - Cathepsin B/L (for cardiomyocytes) [48] - TMPRSS2, NRP1, CD209 (for endothelial cells) [53]	- CAR [168] - DAF [169]	- Gb4 [170] - Ku80 [171] - Integrin α5β [172]
Possesses RNA-dependent RNA polymerase	Yes [68]	Yes [173]	No
Creates DMVs for viral replication	Yes, closed conformation with molecular pores [86]	Yes, open vaselike and closed conformation [174]	No
Cytokine activation in myocardium	- TNF-α, IFN-γ, chemokine ligand 5, IL-1β, -6, -7, -8, and -18 [97]	- IL-1β, IL-6, IL-18, TNF-α type I and type II interferons [92,175]	Interferon-γ, TNF-α, IL-6, IL-8 [176]
Dysregulated immune-cell activation	- Dysregulated T-cell response [105] - Infiltration of macrophages and cytotoxic T cells [17] - Increase in T-helper 17 cells [91]	- Infiltration of macrophages and cytotoxic T cells [92] - Increase in T-helper 17 cells [177]	Infiltration of macrophages and cytotoxic T cells [178]
Modulation of cell death	Activates anti- and proapoptotic signaling [115]	Activates anti- and proapoptotic signaling [113]	So far, only proapoptotic signal activation in later stages has been reported [179]
Energy metabolism	- Altered mitochondria morphology [84] - Dysregulated respiratory chain complex expression [135,137] - Elevated ROS production [141]	- Mitochondrial fragmentation [130] - Dysregulated OXPHOS complex activity [128] - Elevated ROS production [128]	- Dysregulated respiratory chain complex expression [180] - Downregulation of ROS removing enzyme superoxide dismutase 2 [180]
Viroporin	E and Nsp3a viroporins: Ca^2+^, K^+^, and Na^+^ conducting [101,151,154,156]	2B viroporin: Ca^2+^ conducting [181]	NA

## Data Availability

Data sharing not applicable.
